# Centrifugal Force-Driven Modular Micronozzle System: Generation of Engineered Alginate Microspheres

**DOI:** 10.1038/s41598-019-49244-4

**Published:** 2019-09-04

**Authors:** Sung-Min Kang, Go-Woon Lee, Yun Suk Huh

**Affiliations:** 1 Department of Biological Engineering, Biohybrid Systems Research Center (BSRC), Inha University, 100 Inha-ro, Incheon, 22212 Republic of Korea; 2 The Wallace H. Coulter Department of Biomedical Engineering, Georgia Institute of Technology and Emory School of Medicine, Atlanta, Georgia 30332 United States; 3 Platform Technology Laboratory, Korea Institute of Energy Research (KIER), 152, Gajeong-ro, Daejeon, 34129 Republic of Korea; 4 WCSL of Integrated Human Airway-on-a-Chip, Inha University, 100 Inha-ro, Incheon, 22212 Republic of Korea

**Keywords:** Biomedical engineering, Biomaterials

## Abstract

In this study, we developed a modular micronozzle system that can control the flow of fluid based on centrifugal force and synthesize functional alginate microspheres with various structures and sizes. Our method is to fabricate a programmable microreactor that can be easily manufactured without the conventional soft-lithography process using various sequences of the micronozzles with various inner diameters. To overcome the obstacles of pump-based microfluidic devices that need to be precisely controlled, we designed the programmable microreactor to be driven under centrifugal force with a combination of micronozzles, thus enabling the mass production of various functional alginate microspheres within a few minutes. The programmable microreactor designed through the arrangement of the modular micronozzles enables the formation of various types of alginate microspheres such as core-shell, Janus, and particle mixture. These materials are controlled to a size from 400 µm to 900 µm. In addition, our platform is used to generate pH-responsive smart materials, and to easily control various sizes, shapes, and compositions simultaneously. By evaluating the release process of model drugs according to the pH change, the possibility of drug delivery application is confirmed. We believe that our method can contribute to development of biomaterials engineering that has been limited by the requirement of sophisticated devices, and special skills and/or labor.

## Introduction

The introduction of microfluidic devices in the synthesis of functional materials in the early 21st century is expected to create a new paradigm shift that can overcome the limitations of material synthesis based on traditional chemistry^1–3^. A coin-sized microfluidic reactor can precisely control laminar flow fluids through fine-patterned microchannels, and specific advantages such as perfect mixing reactions in a short time, and heating reactions with little local temperature difference are available^4–6^. Particularly, these microfluidic devices can control the size and various shapes of microdroplets according to the channel design and are sufficiently attractive to accept new synthesis techniques^7,8^. For example, polydimethylsiloxane (PDMS)-based flow-focusing microfluidic reactors were based on the droplet pinch-off in immiscible flows at a geometrical complex channel design^9,10^. Because of the surface-tension phenomenon, such methods can be performed to generate various types of functional materials that are monodisperse single spheres as well as core-shell, Janus, and multiple emulsions^11,12^. However, the research trends of microfluidic reactors over the past decade show that they are relatively distant from the expectations of early researchers.

A clear gap exists between the current microfluidic technologies that can meet the recent materials trends, despite the considerable effort expended to realize the creation of new functionalities with highly controlled physicochemical parameters such as size, shape, composition, and surface property for the extension of specified applications using custom-built microfluidic reactors^13,14^. This could be due to the high costs associated with microfluidics, and the requirement for well-trained skillful labor^15–17^. This means that the microfluidic reactor must be made inexpensively and quickly in accordance with the design of various materials, to satisfy customers who demand prototype results in a short time^18^. As shown in the recent works, microfluidic reactors have been developed using glass, silicon, and PDMS polymers based on lithographic processes^19–21^. Hence, it is a passive manufacturing process to integrate the microchannels and align the center of the inner microchannel, which is an important process in manufacturing a typical flow-focusing reactor for synthesizing a functional material. Additionally, the microfluidic reactor should overcome the limitations of traditional methods of transporting fluids in microchannels using external pressure and electric fields^22,23^. Generally, a microfluidic reactor requires a microsyringe pump to tune the fluid flow direction and regulate the adjusted volumetric flows^24^. In particular, it takes a relatively long time to derive such a steady-state flow of a variety of viscous fluids by inducing conventional pumping methods, and has limitations to connect to a continuously expandable mass-production of a wide range of customized smart materials^25–27^. Hence, a limit exists in the demand for quickly changing the device design and implementing customized prototype materials according to various material designs^28–30^. Therefore, the development of end-user friendly microfluidic techniques using simple, easy, and cost-effective methods remains a challenge in the design of a variety of functional materials with monodisperse and narrow size distribution without any deformation^31–33^.

Previously, Takeuchi *et al*. have reported the centrifuge-based droplet shooting device (CDSD) for the generation of 3D multi-compartmental particles using a multi-barrelled capillary^34^ and Kim *et al*. demonstrated the sensory polydiacetylene (PDA) liposome encapsulated multi-phasic alginate microparticles generation by using a custom-built needle injection system^35^. Although the centrifuge-based system has successfully been presented to generate the complex alginate microparticles, these techniques are still arrowed irreversible device fabrication procedure which limits their flexibility for disassembly process.

Herein, we present a novel method to generate engineered alginate microspheres using a centrifugal-force-driven modular micronozzle system. This strategy is very attractive in that the micronozzle device preparation and operation are very easy and convenient, requiring no complicated skill but just a centrifuge and the easy combined micronozzle system. In addition, this strategy is not only useful to generate a complex alginate microsphere, fast, and reproducible manner, but can also yield scalable production. Depending on the shape and size of the engineered alginate microsphere, our programmable micro-reactor is customized by different combinations of microcentrifuge tube, microneedle, and conical tube, which are generally easily found in the laboratory. This system was operated within a few minutes by centrifugation-induced gravitational force. In addition, we show the modular micronozzles with different sequences that can be made with predetermined geometries including core-shell, Janus, and mixtures of size-controlled individual particles. Finally, we investigate the potential application in smart drug delivery systems using simultaneously generated pH-responsive model functional medicines. We envision that this universal strategy may serve as an on-demand platform for a wide range of real applications, especially for the development of advanced smart materials in biomedical engineering with new functionalities.

## Results and Discussion

### Generation of engineered alginate microspheres

**Figure 1:**
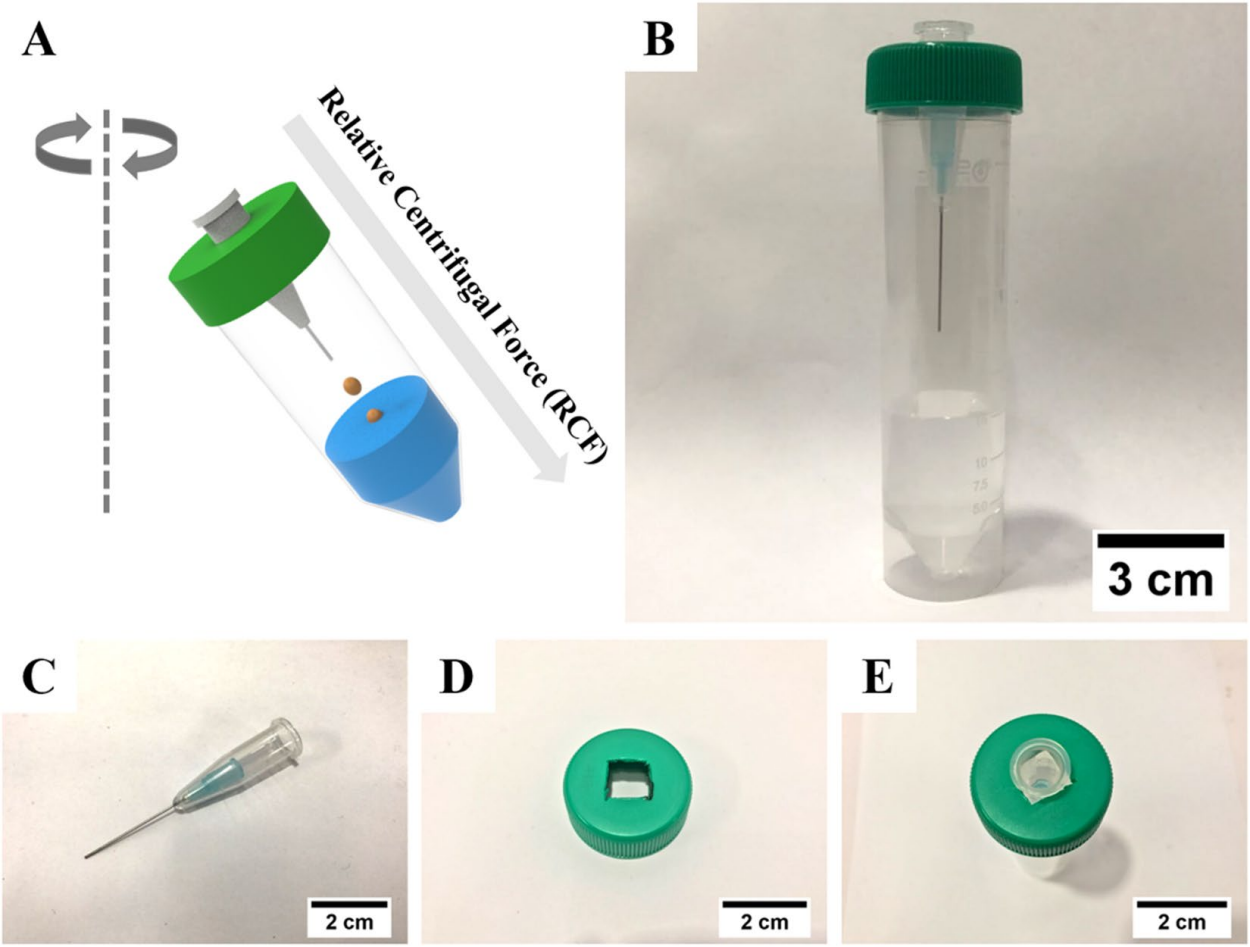
Centrifugal force-driven modular micronozzle system setup. (**A**) A schematic of the basic concept for generation of alginate microsphere. (**B**) A photo of centrifugal microfluidic device after assembled. The modular micronozzle consists of (**C**) a micronozzle and (**D**) a supporter. (**E**) A photo of assembled modular micronozzle.

The implementation of a material-customized changeable reactor is key for determining specific individual functions to apply to various applications^36–39^. Figure 1 shows a schematic illustration of the modular micronozzle design for the generation of complex alginate microspheres. Our approach is based on an external gelation called the *in-situ* dripping direct method which is directly induced the alginate gelation without diffusion control^40^. Further, it is possible to fabricate programmable modular microreactors that are coded with the structure and shape of the alginate through a simple combination of micronozzles. In addition, to overcome the limitation of the conventional pump-based microreactor, which requires careful fluid control, a user friendly microreactor was implemented by inducing fluid flow using centrifugal force (Table S1). This centrifuge-based easy setup is composed of three functional parts: a modular micronozzle, a micronozzle supporter, and a collection tube (see Fig. 1B–E). The desired functional materials were produced within 3 min through the programmable modular microreactor, in which the size and shape of the alginate particle were coded according to the assembly of the micronozzles having different inner diameters. More specifically, the alginate microsphere generation mechanism was followed by a “quasi-static droplet formation”, where a breaks alginate solution off immediately the centrifugal gravity force-induced drag force (*F*_*g*_) exceeds surface tension-induced counter acting force (*F*_*s*_). Based on this, the alginate microspheres were generated from different modular micronozzles when a higher centrifugation was conducted than the surface tension of the alginate aqueous solution.

**Figure 2:**
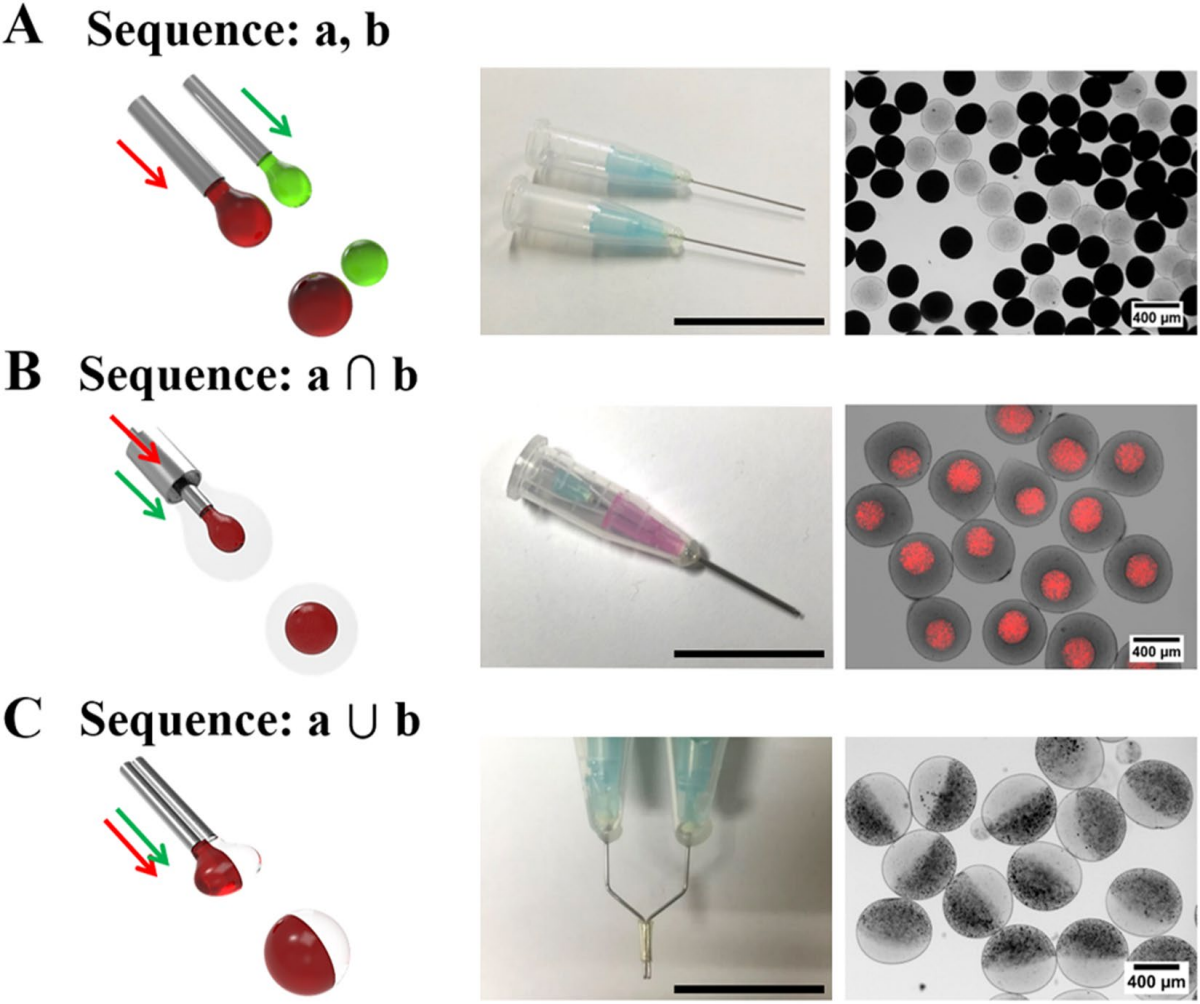
Generation of complex alginate microsphere. The shape of alginate microsphere is determined by changing sequences of modular micronozzle including (**A**) “a, b”, (**B**) “a ∩ b”, and (**C**) “a ∪ b”, respectively. The scale bars represent 3 cm.

Figure 2 shows the modular micronozzle reactors that are programmed to generate the alginate particles of various shapes and sizes through a combination of two needles, *i.e*., independently parallel nozzles, nozzle in nozzle, and union of two nozzles. The first micronozzle was designed to simultaneously synthesize two alginate particles containing different functional particles and sizes as shown in Fig. 2A. Two micronozzles, named sequence “a, b”, were aligned in parallel to the support and mounted in a centrifuge; subsequently, two different composite microspheres were generated within a few minutes. Centrifugal force-driven gravity was induced to release each alginate solution that regularly split to form a spherical droplet. Importantly, the formation of droplets occurred in the regime of water droplets. Then, the droplets passed through the collecting tube, and the inogelation proceeded with the diffusion of the cross-linking agent (i.e., Ca^2+^ ions) into the droplets^41^. The optimal centrifuge duration was determined to be 3 min and was closely related to the conditions under which all of the alginate solution was used. The resulting alginate microspheres showed a spherical shape with an average mean diameter of 372.53 ± 5.68 μm, and a high monodisperse feature (C.V. = 1.52%).

The second proposed micronozzle, named sequence “a ∩ b”, was fabricated to produce the core-shell microspheres by inserting one nozzle into another large diameter nozzle, as shown in Fig. 2B. A small needle can be used to deliver the core alginate fluid, while a relatively large round needle surrounding the small needle can be used for delivering the shell alginate fluid; the resulting alginate droplets are formed at the core-shell structure. To create uniformly sized and well-organized core-shell alginate microspheres, it is important that the center alignment of the small syringe needle be accurately equipped in the opening of the large round needle, and subsequently set using epoxy resin. In addition, the inserted center needle protruded 1–2 mm from the surrounding needle opening (Fig. S1A). Using this micronozzle assembly, the smaller core is efficiently encapsulated during centrifugation as a droplet generation. Subsequently, two different concentric alginate solutions, of high and relatively low concentrations, were introduced in the surrounding and center-located plastic tubes, respectively (Fig. S1B).

Similarly, 2.0 μm of fluorescent polystyrene (PS) microsphere, as an indicator, can be loaded into the core of the alginate microspheres and identified using fluorescence microscopy image analysis, as shown in Fig. 2B. Although the same material was used, the difference between the core and shell interface can be clearly observed: the majority of fluorescence dye is encapsulated in the core, while almost no fluorescence signals can be found in the shell. This approach allows us to readily generate complex core-shell microspheres. In addition, multicompartment core-shell alginate microspheres were generated by sequential centrifugation repeating method which is followed by two-step procedures such as small core particle generation (1^st^ step) and shell generation using core particle distributed alginate solution (2^nd^ step) (Fig. S2).

This simple approach is applicable to the formation of Janus-type complex microspheres to modify the modular micronozzle. The third designed micronozzle, named sequence “a ∪ b”, was designed to combine the Y-shaped modular micronozzles to synthesize the Janus alginate microsphere (Fig. 2C). The bright-field image clearly indicated that the distinct spatial separation in the alginate microsphere was successfully induced after centrifugation. Before the generation of Janus alginate microspheres, two different alginate solutions, *i.e*., an original alginate solution and magnetic nanoparticles (Fe_3_O_4_) dispersed in the alginate solution, were prepared and injected into the combined Y-shape micronozzles that were penetrated into the micronozzle supporter (Figs S1C and S1D). Moreover, the resulting Janus alginate microspheres showed quick response to the static magnetic field, as shown in Fig. S3. This result suggests that our novel strategy can be applied to create dual functional materials such as stimuli-responsive smart materials and microactuators^42,43^. Furthermore, these results suggest the significance of controlling both the shape and chemical heterogeneity of microspheres to extend their potential applications.

### Effect of control parameters for generation of alginate microspheres

**Figure 3:**
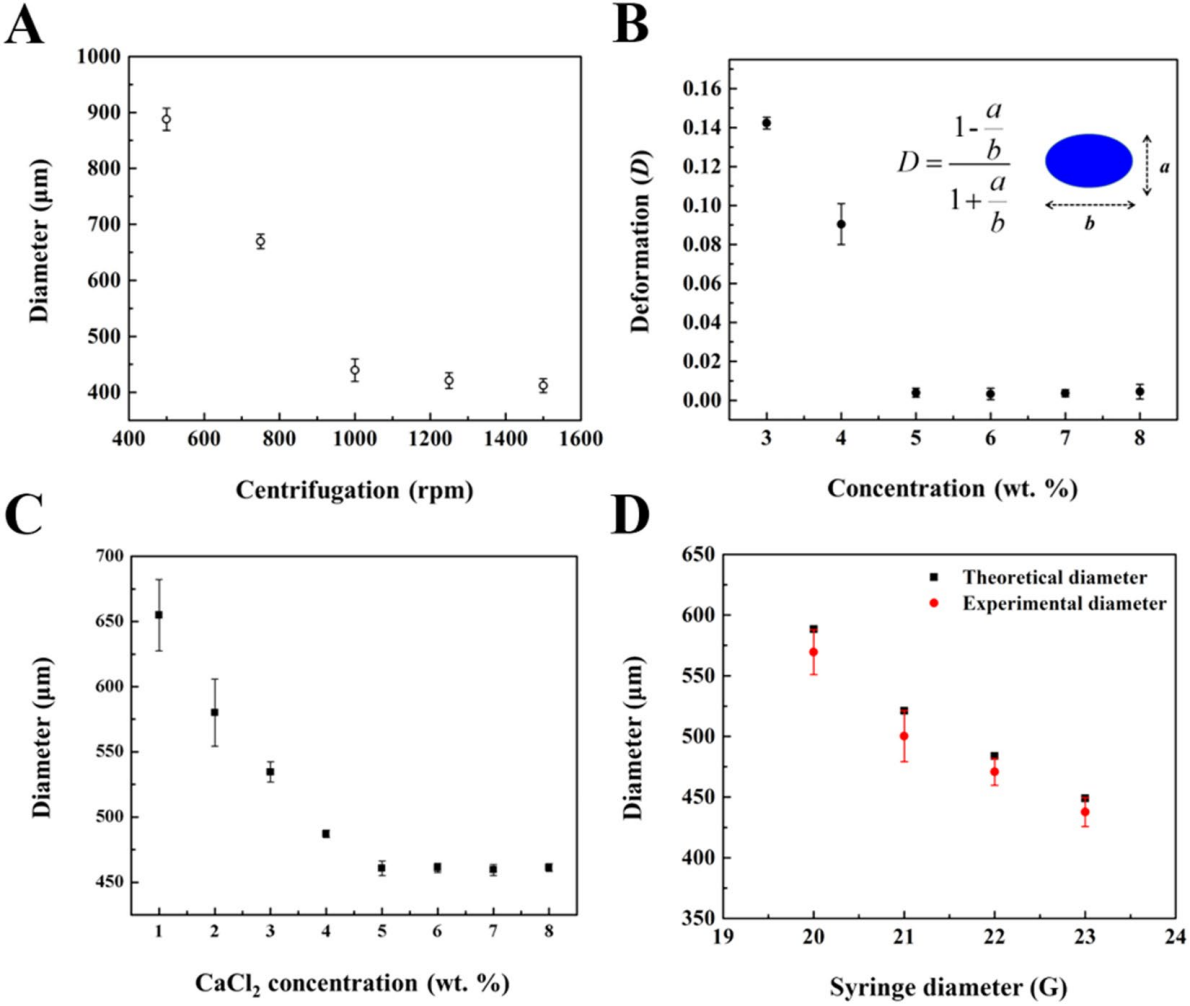
General trend for generation of alginate microsphere. Relationship between physical characteristic and control parameters such as (**A**) centrifugation speed, (**B**) alginate concentration, (**C**) CaCl_2_ concentration, and (**D**) syringe diameter. Each experiment was repeated five times.

To understand the effect of various parameters for the generation of alginate microspheres in our system, we conducted control experiments by varying the conditions such as the centrifugation speed, alginate and CaCl_2_ concentrations, syringe diameters, and distance between the micronozzle tip and the surface of CaCl_2_ solution. These parameters primarily affect the size, shape, and quality of the produced alginate microspheres. First, we generated the alginate microspheres at various centrifugation speeds (*rpm*), while maintaining the alginate concentration at 5 wt.% to examine the relationship between *rpm* and microsphere size, as shown in Fig. 3A. Interestingly, monodisperse microspheres were formed by increasing the centrifugation speed. This strongly suggests the formation of droplets in the dripping regime. In addition, the centrifugation speed is closely related to the driving force of the alginate solution flow, which affects the size of the alginate microspheres as shown in Fig. S4. The bright-field images clearly show that the size of the microspheres was gradually decreased by increasing the rpm. The alginate microspheres with a narrow size distribution was formed using 400 < *rpm* < 2000; outside this range of centrifugation speed, the deformation of alginate microspheres was induced. By changing the speed value from 500 to 1500 rpm, we obtained alginate microspheres with a broad size from 412.01 ± 12.30 to 887.84 ± 19.86 μm. Thus, the centrifugation speed primarily affects the size of the microspheres, and the optimal condition was determined as 1000 rpm based on the C.V. values being less than 3%. As shown in Fig. 3B, the deformation of the alginate microspheres was carefully measured as a function of alginate concentration (*C*_*alginate*_). To define the deformation, the dimensionless deformation (*D*) of the microspheres is as shown in Eq. (1)^34^.

1 $$D=\frac{b-a}{b+a}$$

where *a* and *b* signify the minor and major axes of the synthesized microspheres, respectively. With different concentrations used from 3 to 8 wt.%, the deformation parameter is decreased and saturated, indicating that the microspheres are transformed from an ellipsoid to a sphere (Fig. S5). Moreover, elaborate size control can be conducted by varying the concentration of Ca^2+^ ions (C_ca2+_). When the Ca^2+^ ion was increased from 1 wt.% to 8 wt.%, and the other parameters were maintained as constant (*i.e*., *C*_*alginate*_ = 5 wt.%, *rpm* = 1000, and *G* = 23), the size of the alginate microsphere was decreased from 654.86 ± 27.42 to 460.72 ± 5.48 μm (Figs 3C and S6). This result can be clarified by the formation of dense alginate polymer networks resulting from fast ionic crosslinking that occurred at a high concentration of Ca^2+^ ion, thus yielding a strong ionic binding force between COO^−^ and Ca^2+^ ions. Finally, we investigated the relationship between microsphere size and syringe inner diameter (*G*). To examine the effect of *G*, needles of different gauges were used while retaining all other optimized conditions (*i.e*., *C*_alginate_ = 5 wt.%, *C*_Ca2+_  = 5 wt.%, and *rpm* = 1000). The achievable size of the microspheres ranges from 437.68 ± 11.85 to 569.50 ± 8.49 μm using commercially available different-gauged needles (Figs 3D and S7). Importantly, the gauge-controlled generation of the alginate microsphere could be easily estimated using Eq. (2) that describes the balance of the forces acting on the pendant drop detached from the modular micronozzle^44^.

2 $$d={a}^{3}\sqrt{\frac{{d}_{0}}{g}}$$

where *d* and *d*_0_ are the diameters of the final alginate droplet and the modular micronozzle, respectively; *g* represents the centrifugal force driven gravity; *a* is the coefficient value comprising the surface tension of alginate and the micronozzle circumference. Comparing the experimental and theoretical value, the size difference confirmed the shrinkage of the alginate microsphere during ionogelation. From the comparison between the control experiment and theoretical approach, we obtained the primary control parameters for the generation of the alginate microspheres.

For the investigation of the distance between the nozzle tip and the surface of CaCl_2_ solution, we have conducted experiment with different distances of micronozzle reactor by changing the height of CaCl_2_ solution while keeping all other conditions (*i.e*., *C*_alginate_ = 5 wt.%, *C*_Ca2+_  = 5 wt.%, and *rpm* = 1000). As shown in Fig. S8, the distance (*L*) between the micronozzle tip and the surface of CaCl_2_ solution is an important parameter for the evolution of alginate shape. When the micronozzle tip is closed and soaked in the CaCl_2_ solution (*i.e*., *L* *≤* 1 cm), the shape of fiber can be generated because of the inhibition of surface tension and quick gelation upon facing the CaCl_2_ solution. Following to a previous work by Takeuchi^34^, we can also find criteria for the evolution of alginate in our system. Thus, the optimal condition was determined as 3 cm (*L* > 1 cm) regarding to generate the alginate microspheres. Furthermore, the shape and size control can be accomplished by simply assembling the optimized control parameters although the same device was used repeatedly.

### One-step generation of different sized alginate microspheres

The ultimate goal of this study is to develop a simple system with a universal modular micronozzle design for easy use in academic and practical applications. Among the physical parameters, the particle size, in particular, determines the functionality of that particle such as its uptake, adherence, degradation, as well as residence in circulation^45,46^. In addition, constant release rate (*i.e*., ‘zero-order‘ release) is highly ideal for the drug delivery system which can be achieved by mixing various sized microspheres^47–50^.

**Figure 4:**
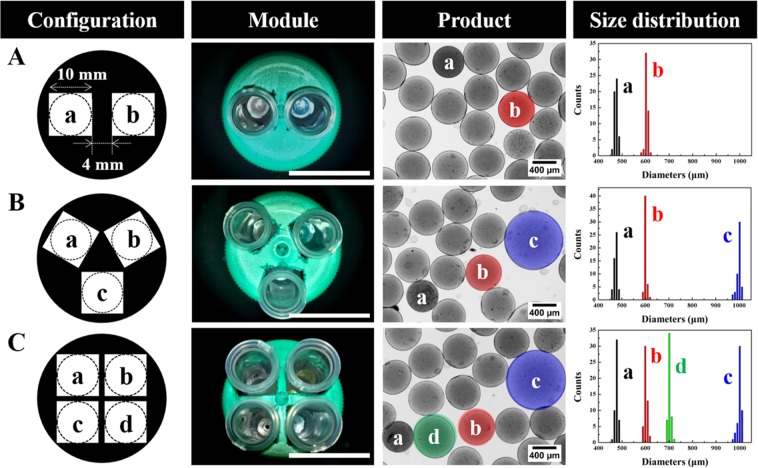
Configuration of modular micronozzle using different micronozzles. One-step generation of a broad ranged microspheres with various configurations such as (**A**) “a-b”, (**B**) “a-b-c”, and (**C**) “a-b-c-d”. The scale bars represent 3 cm. Each experiment was repeated five times.

Thus, the next challenge for confirming our accessibility is to generate different sized alginate microspheres using a modified modular micronozzle with one-pot generation. As shown in Fig. 4, we designed and created an array of modular micronozzles comprising different numbers of square holes on a micronozzle supporter, to generate the different size-controlled alginate microspheres simultaneously. For example, the modular micronozzle consists of two different gauged needles, called the “a-b” configuration herein, and can generate two types of alginate microspheres, as shown in Fig. 4A. The corresponding bright-field images demonstrated the “particle mixture” containing various sizes of the alginate microsphere. The parallelized configurations with different gauged needles were affected by the equal gravitational force through centrifugation, thus enabling various sizes of alginate microsphere to be generated. Figure 4B,C show the general hierarchical trend of the one-pot synthetic procedure for the different-size microsphere generation, and the number of alginate microspheres well-matched with the number of different micronozzles using various configurations such as “a-b-c” and “a-b-c-d,” respectively. For example, the average sizes of alginate microsphere using “a-b-c-d” configuration, which consist of 22 G, 20 G, 17 G, and 19 G, were calculated as 487.11 ± 3.60 μm, 603.78 ± 4.10 μm, 1039.72 ± 12.60 μm, and 703.72 ± 6.60 μm, respectively. Since the alginate microspheres were generated using the modular micronozzle consists of different gauged needles, the particles showed a similar trend with single micronozzle generation (Fig. S9). In addition, the results show the narrow size distributions, and that the C.V. of the alginate microspheres generated in each modular micronozzle is less than 3%. This novel strategy has demonstrated the potential for the one-pot generation of engineered microspheres with precise size control in a broad range.

### Application for drug delivery system

**Figure 5:**
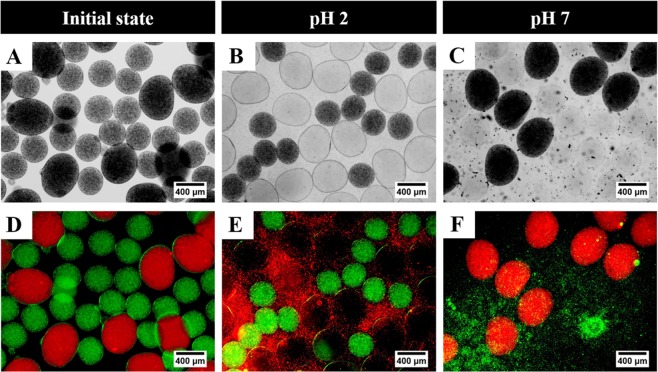
Model system for possible application to drug delivery system using pH-responsive alginate microspheres encapsulating green and red fluorescence PS microsphere. Bright-field images of different alginate microspheres to apply different pH at (**A**) initial, (**B**) 2, (**C**) 7 and (**D**–**F**) its corresponding fluorescence images. The external temperature was maintained at 37 °C.

To evaluate the practicability of our proposed device, we synthesized the stimuli-response functional particles assuming drug delivery in the body. Stimuli-response microspheres have attracted particular attention as these materials are a potential candidate for a wide range of “smart” applications by physical stimuli including temperature change, light, and magnetic field, and chemical stimuli such as pH, ionic strength change, and chemical or biological agents^51–53^. In addition, there are various works applied to the use of alginate microspheres as a carrier for the drug delivery system^54–57^. Thus, we examined the potential application of the smart material synthesized through our device as a model for the oral drug delivery system. This system is known to show a drastic change in pH from the stomach (acidic pH 2) to the intestine (basic pH 5–8) in the GI tract, sequentially^54^. As shown in Fig. 5A, we used the modular micronozzle with “a-b” configuration and simultaneously generated two different alginate microspheres containing amino- and carboxyl-functional groups with different concentrations of alginate solution. In detail, the preparation of stimuli-responsive two different particles that is generated by one-pot procedure using “a-b” micronozzle sequence. The particle shape is a significant parameter in drug encapsulation application. Therefore, two micronozzles, such as 21 and 25 G, used for generating pH 2-responsive ellipsoid and pH 7-responsive spherical alginate microspheres simultaneously. It can be attributed for generating various types of drug encapsulated carriers and delivering diverse medications in the human body, especially gastrointestinal tract^58^. To visualize drug releasing, we used artificial components (*i.e*., polystyrene beads) as a model drug, and synthesized various sizes and shape-controlled alginate microspheres for defining the number of polystyrene beads per microsphere (Fig. 5D). The alginate microspheres were subsequently incubated with different pH aqueous solutions. As shown in Fig. 5B and the corresponding fluorescence image of Fig. 5E, the ellipsoidal-shaped microspheres swelled and released red fluorescence tagging the artificial drugs after an incubation time of 12 h at 37 °C. This occurred because the cationic polymers containing amine groups became charged quaternary ammonium salts (-NH_4_^+^), and induced swelling under low pH conditions below the pKa value threshold. On the contrary, relatively small spherical microspheres responded at pH 7 (Fig. 5C,F). The actuation of the swelling equilibrium is important information as it provides the understanding of the interaction between the polymeric networks and transportation, and the degradation of fluid in three-dimensional systems in which a microsphere will challenge at different environments. For example, the carboxyl-group-functionalized microspheres show the non-ionized form (-COOH) and decreased hydration at a low pH of 2. When the pH of the solution increases above the pKa, the carboxylic group changed to the ionized form (-COO^−^), leading to an increase in electrostatic repulsion, thus causing polymer expansion and higher swelling of the alginate polymer matrix, being the highest around pH 7^59^. Apart from the various parameters discussed, the microsphere size, shape, and composition are closely related and contributed to their special function. Comparison between previous centrifuge system, our system can be used as a simple method to design biomaterials which overcome by considering all of important factors to target applications (Table S2).

## Conclusion

We presented a novel approach to a centrifugal-force driven modular micronozzle system, and its simple operation for the generation of complex engineering microspheres. The modular micronozzle device was composed of conventional available laboratory apparatus including syringe needles and centrifuge tube. The device can be turned by adjusting various sequences such as “a, b”, “a ∩ b”, and “a ∪ b”. Interestingly, our novel approach enabled the preparation of 400 µm to 900 µm microspheres with complex geometries (*i.e*., core-shell and Janus), and the generation of pH-responsive smart microspheres with different sizes, shapes, and compositions for applications in smart drug delivery systems.

Despite the recent advances in centrifugal device systems, this study has some innovative points compared to previous reports; (1) We present a highly reproducible and reversible micronozzle reactor for the generation of engineered alginate microspheres. (2) Various combinations of micronozzles, such as nozzle in the nozzle and union assembling, have shown that the structure-controlled alginate particles, such as single, Janus and multi-core shells, are produced reproducibly. (3) The shape and size control can be pre-estimated and demonstrated by changing experimental parameters such as centrifugation speed, alginate and CaCl_2_ concentrations, syringe diameters and distance between micronozzle tip and CaCl_2_ solution. (4) Our modular micronozzle reactor can be mounted on any commercial conventional centrifuge to perform the generation of complex microspheres with simple preparation, low operating costs, and high reproducibility. (5) A large number of microspheres (approximately 1,000 particles per 1 mL alginate solution) can be produced in a few minutes, thus enabling mass production. Therefore, we believe that the combination of both the novel microsphere generation method and automation will create considerable progresses, not only in the real field of drug delivery and tissue engineering fields, but also in smart functional material fields applying microspheres. Using the modular micronozzle system offers several advantages that could be easily assembled, disassembled, reconfigured, and reassembled. In addition, this system would allow to be reversible, simple to use, easy to manufacture and consistent and reliable in their performance following repeated assembly and disassembly process for achieving the large-scale synthesis, cost-effectiveness, higher reproducibility and reusability.

## Methods

### Materials

Sodium alginate, calcium chloride (CaCl_2_), polyvinylpyrrolidone (PVP, Mw = 40,000), polyethylene glycol diacrylate (PEG-DA, Mw = 700), ferric chloride hexahydrate (FeCl_3_·6H_2_O), iron (III) ferrocyanide (PB, Prussian blue, Fe_4_[Fe(CN)_6_]_3_·nH_2_O, n = 14–16), and fluorescein isothiocyanate-dextran (FITC-dextran, Mw = 20,000) were purchased from Sigma-Aldrich Chemicals (MO, USA). Both alginate and calcium chloride solutions were prepared using experimental solutions and further diluted to the required concentrations before use. Milli-Q water having a resistance of less than 18.2 mΩ was used in all procedures.

### Fabrication of centrifugal-force driven modular micronozzle device

The centrifugal-force driven modular micronozzle device was composed of a modular micronozzle, a micronozzle supporter, and a collection tube. These materials were obtained commercially. To fabricate the modular micronozzle, a syringe needle (KOVAX-NEEDLE^®^, Korea Vaccine Co., Ltd., Seoul, Korea) was inserted into the bottom of a 1.5-mL microcentrifuge tube (Eppendorf AG, Hamburg, Germany). A hole was bored by heating up the 23-gauge needle. Subsequently, connections were sealed with epoxy resin to prevent liquid leakage. Subsequently, the micronozzles were inserted into the square holes (i.e., 1 cm × 1 cm) on the tube cap as a micronozzle supporter that can bore a hole, such that the micronozzle would pass through. Finally, a 50-mL centrifuge tube (Becton Dickinson, Cowley, Oxford, UK), as a collection tube, was assembled with screws. Unlike other microfluidic methods, surface treatment was not required in this assembly.

### Generation of single alginate microsphere

The sodium alginate (5 wt.%) solution was extruded through the micronozzle with a 23-gauge syringe needle. The resulting alginate microdroplets were directly corrected in a 5 wt.% CaCl_2_ aqueous solution during centrifugation. To induce the phase contrast under the bright field, the alginate aqueous solutions were prepared by dissolving with/without iron(III) ferrocyanide nanoparticles, separately. In all the experiments, a swing rotor centrifuge (Labogene, Seoul, Korea) was used, and operated at 1000 rpm for 3 min.

### Design of the modular micronozzle for generation of complex microsphere

To produce core-shell alginate microspheres, the modular micronozzle with sequence “a ∩ b” was conducted by inserting the small micronozzles, consisting of a PCR centrifuge tube, located in the center of another big micronozzle. In this study, the different lengths of the syringe needles used for the core formation was 6 cm of the 23-gauge needle (the outer/inner diameters of which were 0.64 mm/0.32 mm), and 3 cm of the 18-gauge needle (the outer/inner diameters of which were 1.27 mm/0.84 mm), to generate the shell. In addition, a Y-shaped modular micronozzle was created by the sequence “a ∪ b” to prepare Janus alginate microspheres. First, two modular micronozzles of the same length (approximately 3 cm) of the 23-gauge syringe needles were equipped to the micronozzle supporter and were subsequently bent two times at 120° to face each other. Finally, it was set by epoxy resin to maintain the Y-shape configuration.

### Generation of pH-response alginate microsphere

pH-responsive microspheres were generated as described above. The amine group functionalized microspheres were prepared by cationic polymers containing alginate aqueous solution (ellipsoid, 40% alginate with 60% PVP). In addition, the carboxyl-group functionalized microspheres were generated by anionic polymers containing alginate aqueous solution (sphere, 40% alginate with 60% PEG-DA). These microspheres were transferred into various pH aqueous solutions and incubated at 37 °C with gentle shaking. To observe the release profiles at different pH environments, we encapsulated two fluorescently labeled PS microspheres inside each alginate microsphere. The microspheres shown in Fig. 5 were visualized by fluorescent microspheres: the compositions of each color are as follows. Red: 0.5 vol. % red-fluorescent PS microspheres (Sigma Aldrich, λ_ex_ ~ 575 nm; λ_em_ ~ 610 nm). Green: 0.5 vol. % fluorescent yellow-green PS microspheres (Sigma Aldrich, λ_ex_ ~ 470 nm; λ_em_ ~ 505 nm).

### Reproducibility test and characterization

The reproducibility of the microsphere generation was verified to prepare complex alginate microspheres using different modular micronozzles (i.e., sequences of “a, b”, “a ∩ b”, and “a ∪ b”). In each case, five independent experiments were conducted. The diameters of the alginate microsphere were determined from the bright-field images captured using a digital camera connected to a microscopy system. The prepared complex alginate microspheres and pH-responsive microspheres were observed with an inverted phase microscope (Olympus IX70, Olympus Optical Co., Tokyo, Japan) in the bright-field and fluorescence modes. The alginate microsphere diameters were determined from images captured using a digital camera (ToupTek Photonics Co., Ltd., China) attached to a microscope. To measure the monodispersity of the alginate microspheres, at least 50 microspheres per batch (1 cycle centrifugation) were evaluated, and the mean average diameters and standard deviations were calculated. Based on this, the coefficient of variation (C.V.) (%) described in the following Eq. (3) was used.

3 $${\rm{C}}.{\rm{V}}.( \% )=\frac{{\rm{\sigma }}}{{{\rm{D}}}_{{\rm{a}}}}\times 100$$

where σ and D_a_ are the standard deviation and the mean average diameter of microsphere, respectively.

## Supplementary information

Supplementary Information
