# Supplementary material for: Centrifugal Force-Driven Modular Micronozzle System: Generation of Engineered Alginate Microspheres
Source: Sci Rep. 2019 Sep 4;9:12776. doi: 10.1038/s41598-019-49244-4 (PMC6726759; doi:10.1038/s41598-019-49244-4)
Supplement: Supplementary file 1 — Supplementary Information [file 41598_2019_49244_MOESM1_ESM.docx]

**Supplementary Information**

**Centrifugal Force-Driven Modular Micronozzle System: Generation of Engineered Alginate Microspheres**

Sung-Min Kang^1^, Go-Woon Lee^1,2^ & Yun Suk Huh^1,3,^*

*^1^ Department of Biological Engineering, Biohybrid Systems Research Center (BSRC), Inha University, 100 Inha-ro, Incheon, 22212, Republic of Korea.*

*^2^ Platform Technology Laboratory, Korea Institute of Energy Research (KIER), 152 Gajeong-ro, Daejeon 34129, Republic of Korea.*

*^3^ WCSL of Integrated Human Airway-on-a-Chip, Inha University, 100 Inha-ro, Incheon, 22212, Republic of Korea.*

* Corresponding authors.

E-mail addresses: [yunsuk.huh@inha.ac.kr](mailto:yunsuk.huh@inha.ac.kr) (Y. S. H.)

Keyword: centrifugal force, modular micronozzle, biomaterials, Janus particle, core-shell

**Estimation of alginate droplet size.** The diameters of final alginate droplet can be calculated using the formula as below.

$Gravity-induced drag force (F_{g})=\frac{\pi\rho d_{p}^{3}g}{6}$ (S1)

$Surface tension-induced force \left( F_{s} \right)=\sigma C=\sigma\left( 2r\pi\right)=\sigma d_{0}\pi$ (S2)

Balance of acting force on the droplet at the detachment from micronozzle ($F_{g}=F_{s}$),

$\frac{\pi\rho d_{p}^{3}G}{6}= \sigma d_{0}\pi$ (S3)

$d=\sqrt[3]{\frac{6\sigma}{\rho}}\cdot\sqrt[3]{\frac{d_{0}}{g}}=a\sqrt[3]{\frac{d_{0}}{g}}$ (S4)

where *d* and *d_0_* are the diameters of the final alginate droplet and the modular micronozzle, respectively; *C* is the circumference of micronozzle; *g* represents the centrifugal force driven gravity; *a* is the coefficient value comprising the surface tension and density of alginate.

In our case, the diameters of alginate droplet can be calculated as follows:

- Surface tension of alginate ($\sigma$) = 66.9 mN/m

- Gravitational force (*g*) = 9.8 m/s^2^

- Syringe inner diameter = 20 G (635 μm), 21 G (559 μm), 22 G (508 μm), 23 G (406 μm)


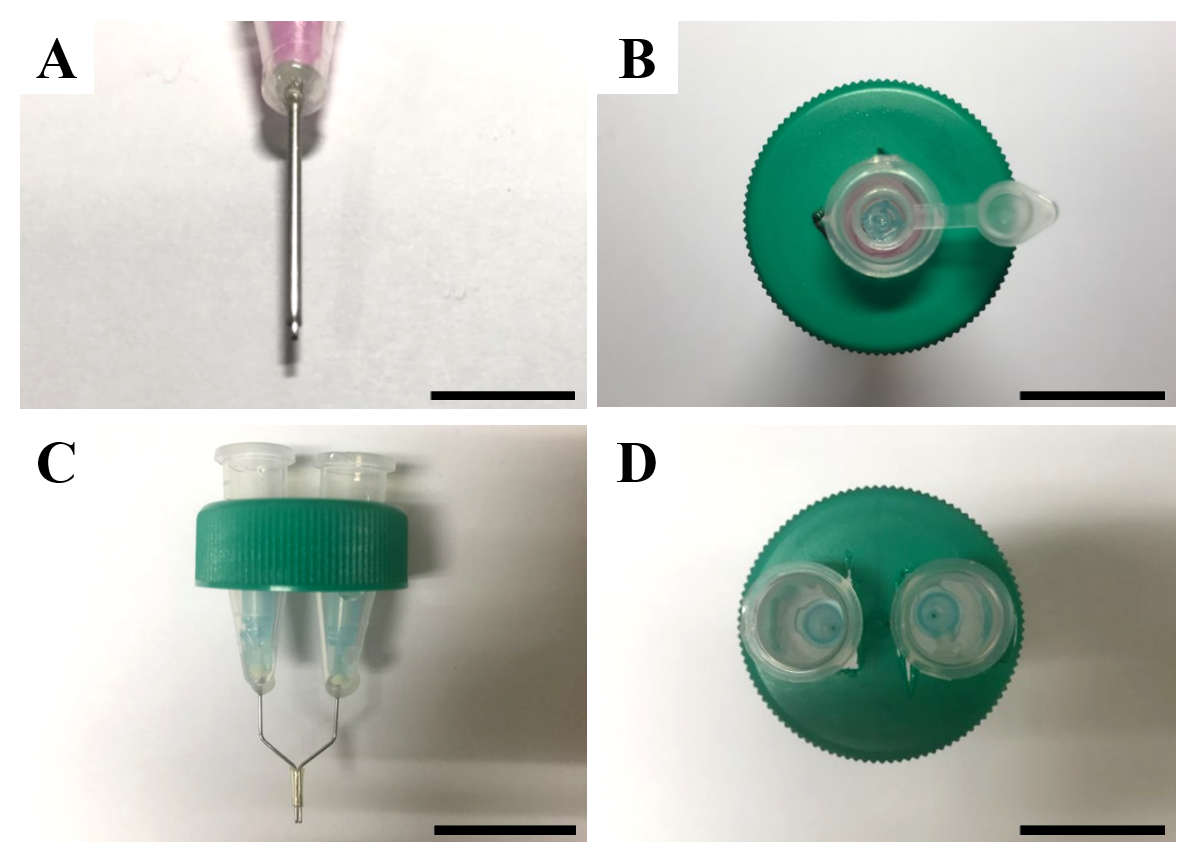


**Figure S****1.** Photographs of the modular micronozzles with sequences. (A and B) “a ∩ b” and (C and D) “a ∪ b”. The scale bars represent 2 cm.


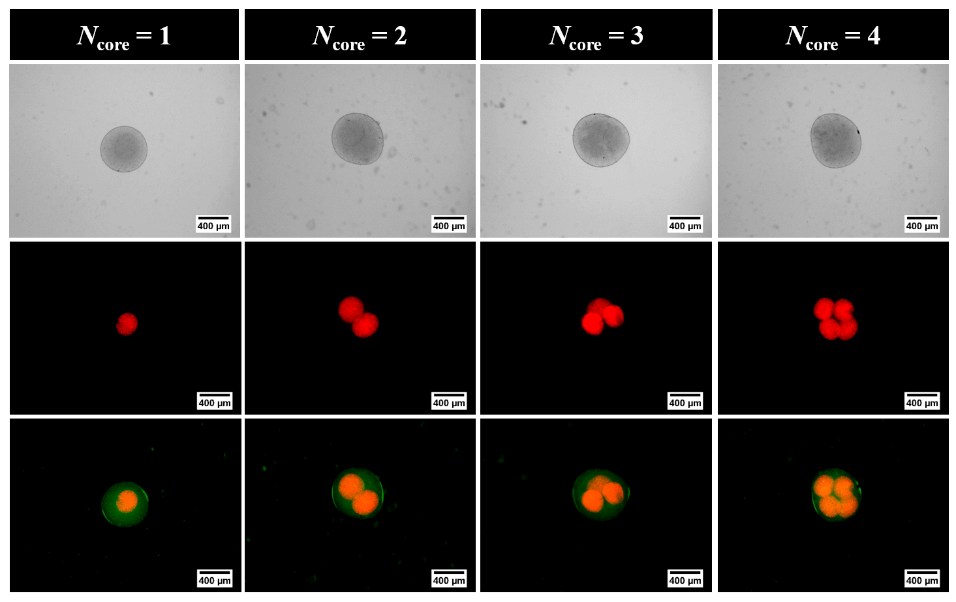


**Figure S2.** Bright-field and fluorescence images of the complex core−shell multicompartmental particles with varying numbers (*N*) of core compartments. Images showing the controlled shapes of the multicompartmental particles by varying micronozzle diameters in the secondary micronozzle device.


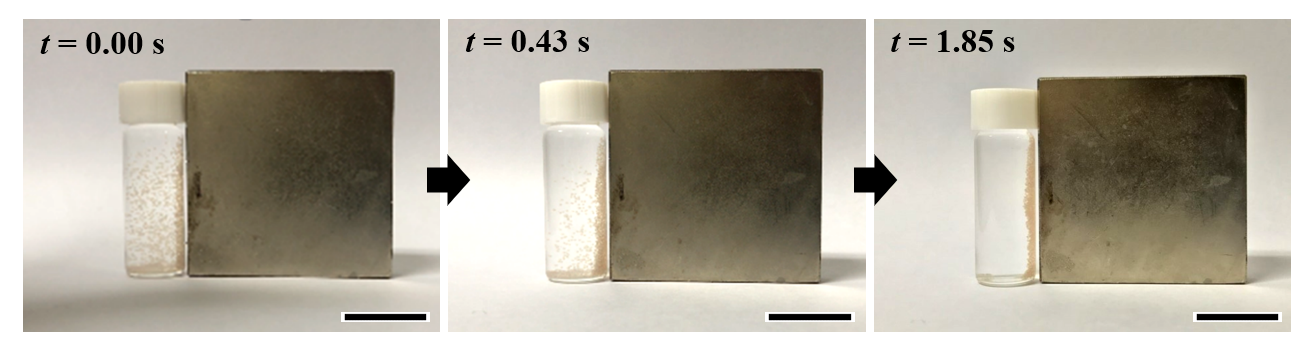


**Figure S3.** Time sequential photographs for quick response behavior of magnetic Janus alginate microspheres to static magnetic field. The scale bars represent 2 cm.


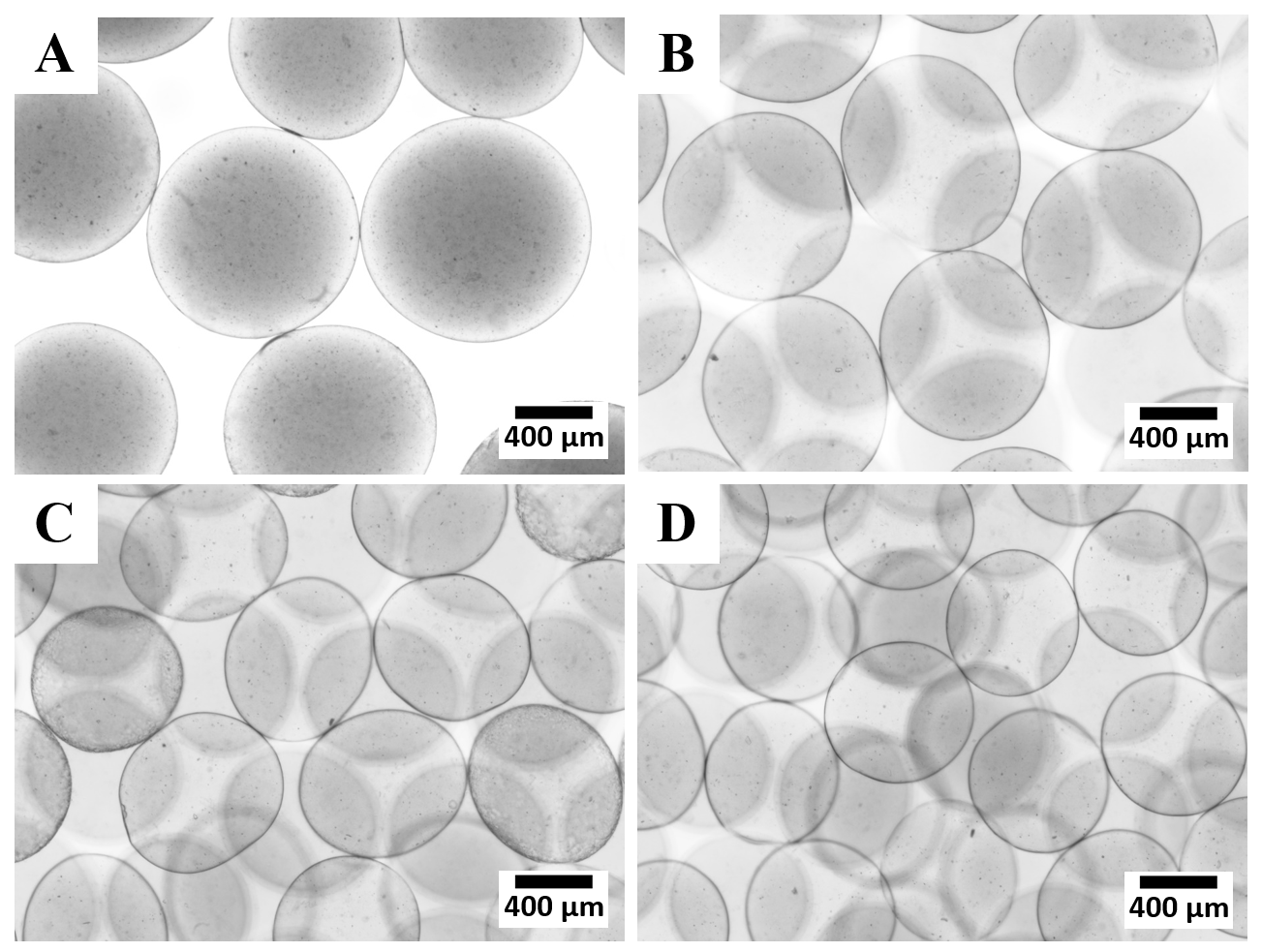


**Figure S4.** Effect of centrifugation speed for generation of alginate microsphere. The size of alginate microsphere was investigated by controlling the centrifugation at (A) 500, (B) 750, (C) 1000, and (D) 1500 rpm. Each experiment was repeated five times.


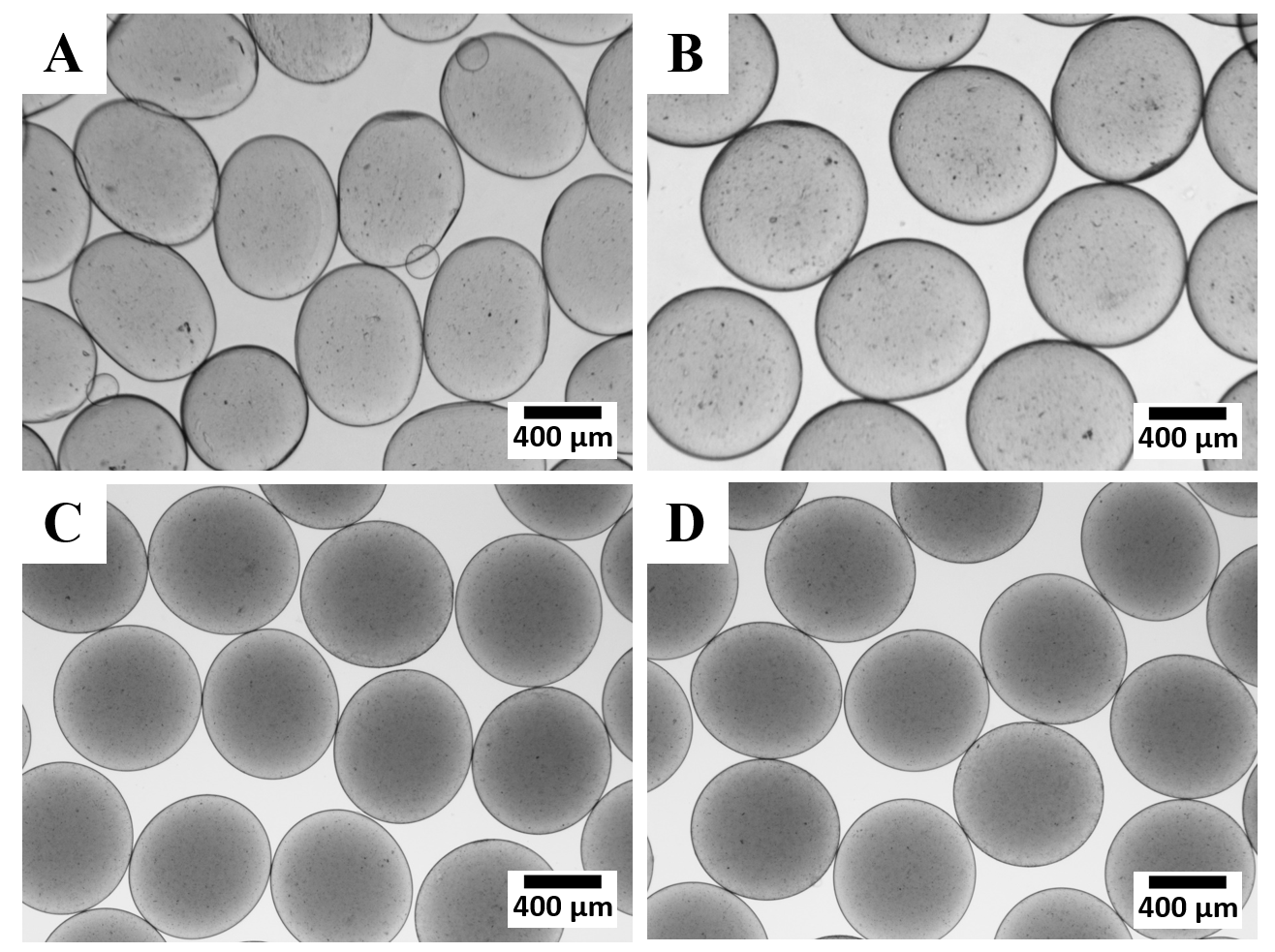


**Figure S5.** Effect of alginate concentration for generation of alginate microsphere. The generated alginate microsphere was investigated by controlling the alginate concentration at (A) 3, (B) 4, (C) 5, and (D) 6 wt.%. Each experiment was repeated five times.


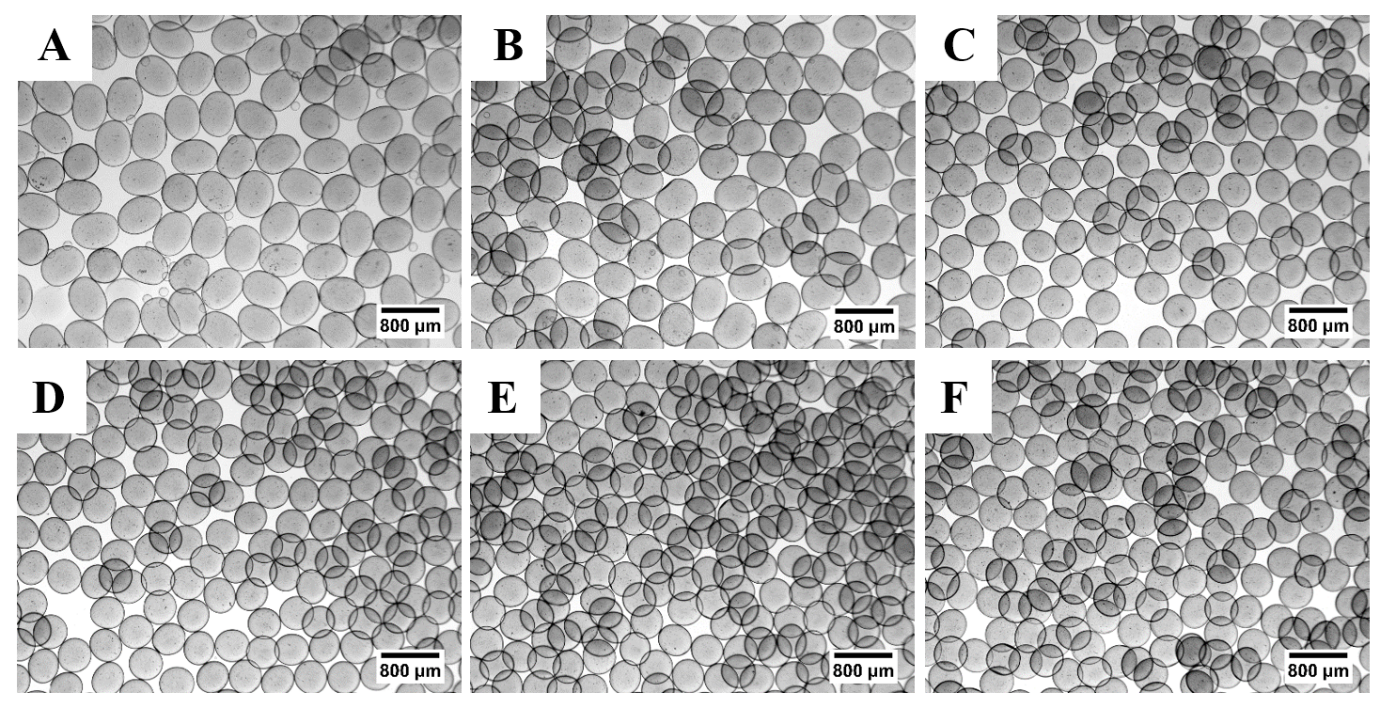


**Figure S6.** Photographs of results for controlling the concentration of CaCl_2_. Different CaCl_2_ aqueous solutions to apply at (A) 3, (B) 4, (C) 5, (D) 6, (E) 7, (F) 8 wt. % respectively. Each experiment was repeated five times.


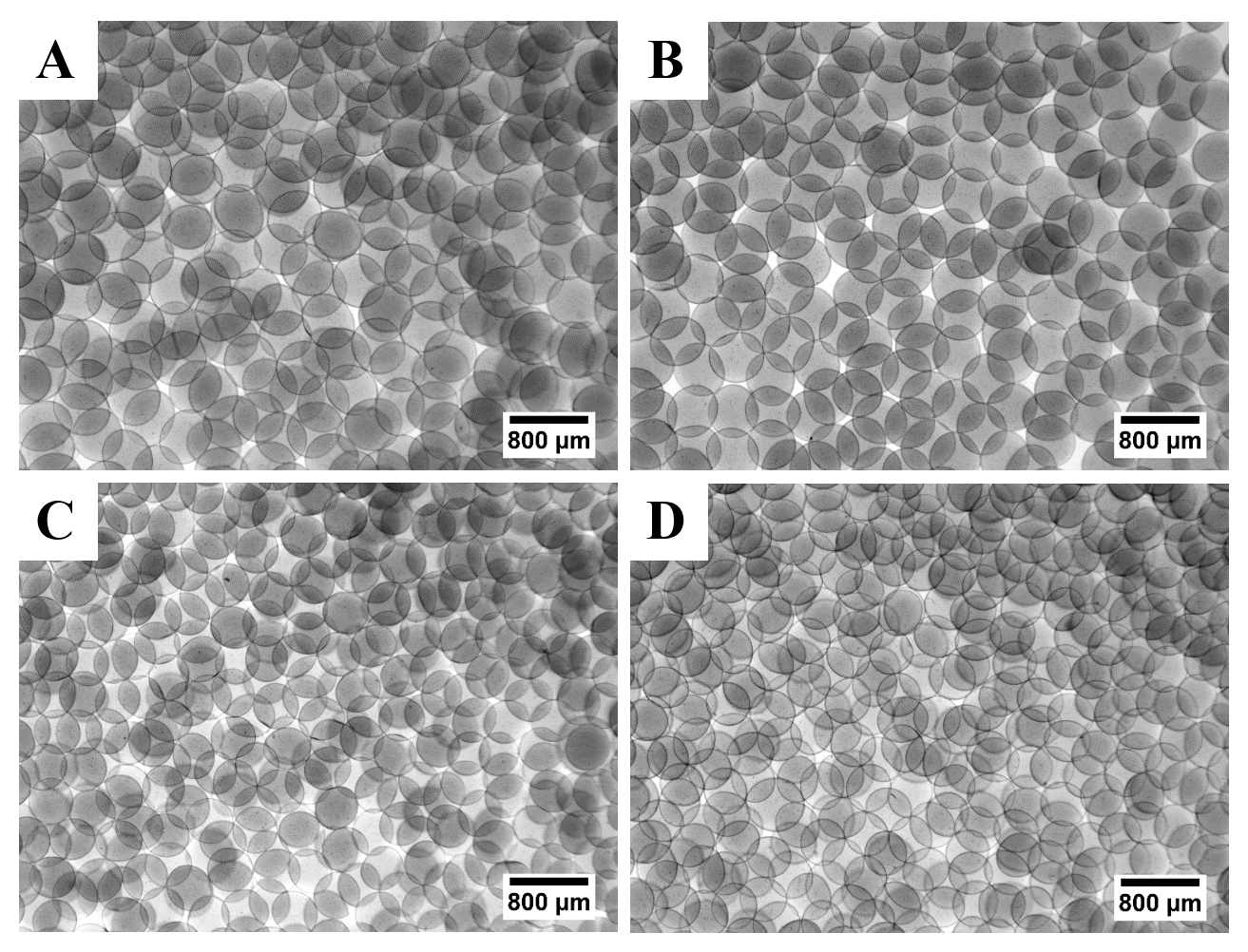


**Figure S7.** Effect of syringe inner diameter for generation of alginate microsphere. The size of alginate microsphere was demonstrated by changing the syringe needle gauge at (A) 20, (B) 21, (C) 22, and (D) 23 G. Each experiment was repeated five times.


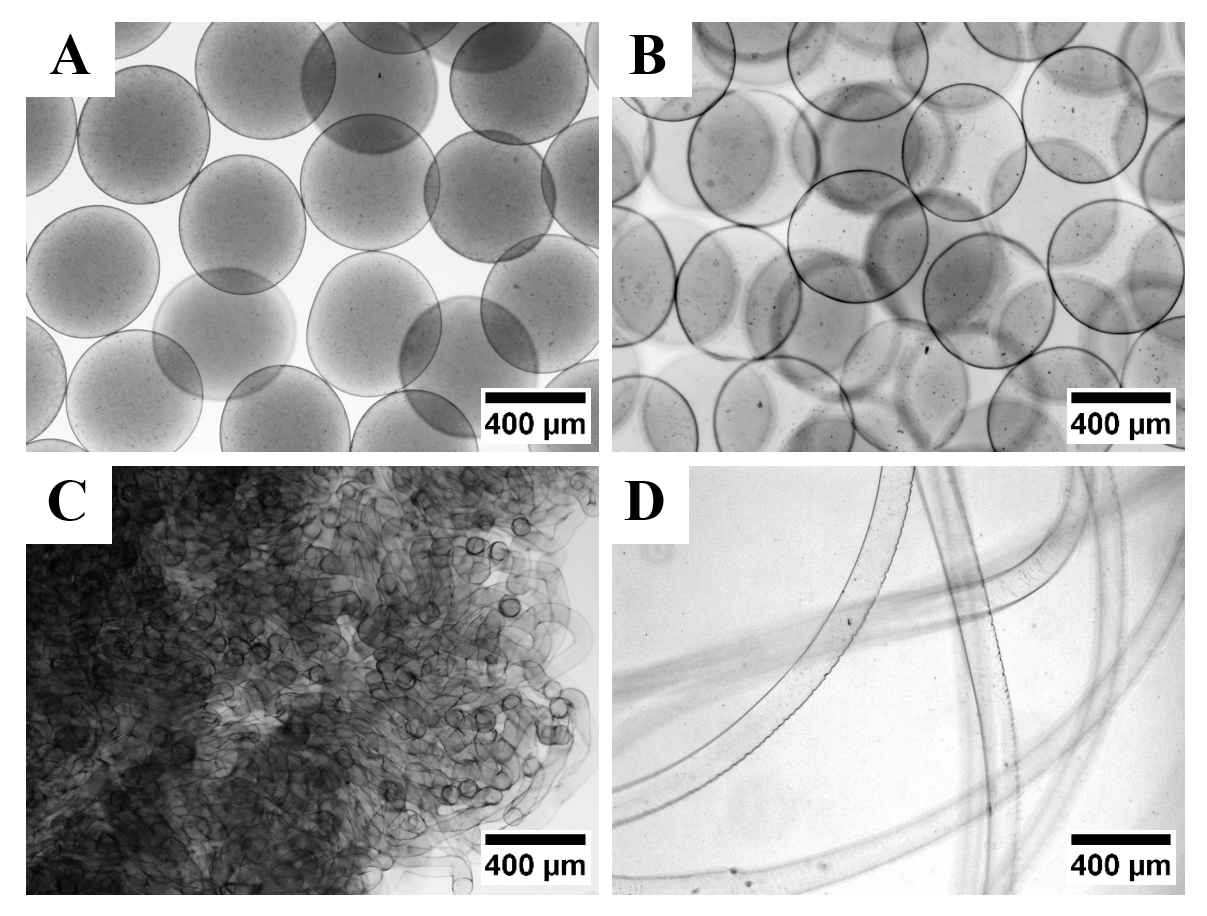


**Figure S8.** General trend for the evolution of alginate shape. Bright field images show the relationship of distance (*L*) between the micronozzle tip and the surface of CaCl_2_ solution at (A) 3, (B) 2, (C) 1, and (D) -1 cm. Each experiment was repeated five times.


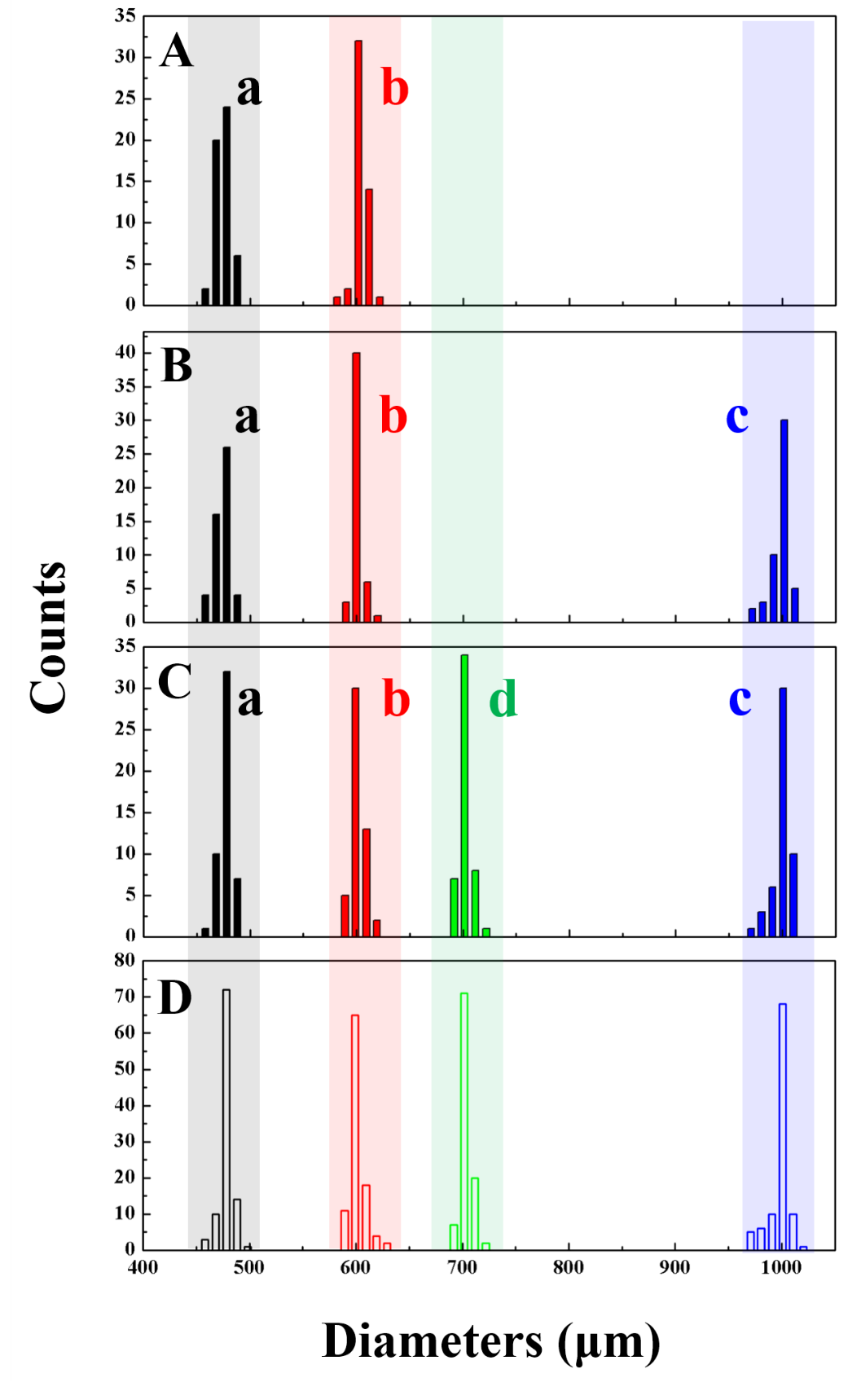


**Figure S9**. Comparison between the size distributions of alginate microspheres using various configurations such as (A) “a-b”, (B) “a-b-c”, (C) “a-b-c-d”, and (D) single micronozzles (“a”, “b”, “c”, and “d”) as a control. Each experiment was repeated five times.

**Table S1**. Comparison between existing glass capillary device and modular micronozzle system to prepare engineered biomaterials.

|  | **Modular micronozzle system** | **Glass capillary device** |
| --- | --- | --- |
| Materials | Needle, eppendorf tube (1.5 ml), conical tube (50 ml) | Cylindrical glass capillary, square glass capillary, needle, tube, epoxy adhesive |
| Equipment for device preparation | Knife (official) | Micropipette puller, microforge, |
| Reproducibility | High | Low |
| Flexibility | High | Low |
| Sample loading | Simple pipetting | Pumping by microsyringe pump |
| Operation | Centrifuge | External pressure by microsyringe pump |
| Handling for end-user | Easy | Difficult |
| Refs. | This work | [1] |

**References**

1.  Kang, *et al*. Microfluidic generation of Prussian blue-laden magnetic micro-adsorbents for cesium removal. *Chem. Eng. J.* **341**, 218-226 (2018).

**Table S2.** Comparison between existing centrifuge system and modular micronozzle system for generation of engineered alginate microspheres.

| **Methods** | **Driving force** | **Reproducibility** | **Reversibility** | **Operation**  **time** | | **Ref.** |
| --- | --- | --- | --- | --- | --- | --- |
| Modular micronozzle system | Centrifugal force | High | Reversible | 5 min | In this study | |
| Centrifuge-based droplet shooting device | Centrifugal force | Middle | Irreversible | 20 s | [2] | |
| Custom-built needle injection system | Centrifugal force | Middle | Irreversible | 1 h | [3] | |

**References**

2. Maeda, K. Onoe, H. Takinoue, M. & Takeuchi, S. Controlled synthesis of 3D multi‐compartmental particles with centrifuge‐based microdroplet formation from a multi‐barrelled capillary. *Adv. Mater.* **24**, 1340-1346 (2012).

3. Lee, J. & Kim, J. Multiphasic sensory alginate particle having polydiacetylene liposome for selective and more sensitive multitargeting detection. *Chem. Mater.* **24**, 2817-2822 (2012).
